# An Analysis of Combined Molecular Weight and Hydrophobicity Similarity between the Amino Acid Sequences of Spike Protein Receptor Binding Domains of Betacoronaviruses and Functionally Similar Sequences from Other Virus Families

**DOI:** 10.3390/microorganisms12102021

**Published:** 2024-10-05

**Authors:** Jamie D. Dixson, Lavanya Vumma, Rajeev K. Azad

**Affiliations:** 1 Department of Biological Sciences and BioDiscovery Institute, University of North Texas, Denton, TX 76203, USA; jamiedixson@my.unt.edu; 2 Texas Academy of Mathematics and Science, University of North Texas, Denton, TX 76203, USA

**Keywords:** physicochemical, recombination, horizontal gene transfer, convergence, twilight zone, Coronavirus, Arterivirus, Astrovirus, Reovirus, Torovirus

## Abstract

Recently, we proposed a new method, based on protein profiles derived from physicochemical dynamic time warping (PCDTW), to functionally/structurally classify coronavirus spike protein receptor binding domains (RBD). Our method, as used herein, uses waveforms derived from two physicochemical properties of amino acids (molecular weight and hydrophobicity (MWHP)) and is designed to reach into the twilight zone of homology, and therefore, has the potential to reveal structural/functional relationships and potentially homologous relationships over greater evolutionary time spans than standard primary sequence alignment-based techniques. One potential application of our method is inferring deep evolutionary relationships such as those between the RBD of the spike protein of betacoronaviruses and functionally similar proteins found in other families of viruses, a task that is extremely difficult, if not impossible, using standard multiple alignment-based techniques. Here, we applied PCDTW to compare members of four divergent families of viruses to betacoronaviruses in terms of MWHP physicochemical similarity of their RBDs. We hypothesized that some members of the families Arteriviridae, Astroviridae, Reoviridae (both from the genera rotavirus and orthoreovirus considered separately), and Toroviridae would show greater physicochemical similarity to betacoronaviruses in protein regions similar to the RBD of the betacoronavirus spike protein than they do to other members of their respective taxonomic groups. This was confirmed to varying degrees in each of our analyses. Three arteriviruses (the glycoprotein-2 sequences) clustered more closely with ACE2-binding betacoronaviruses than to other arteriviruses, and a clade of 33 toroviruses was found embedded within a clade of non-ACE2-binding betacoronaviruses, indicating potentially shared structure/function of RBDs between betacoronaviruses and members of other virus clades.

## 1. Introduction

To date, three deadly betacoronaviruses have affected the human population. Those are SARS-CoV, MERS-CoV, and SARS-CoV-2 [1,2,3,4,5]. SARS-CoV-2, the causative agent of COVID-19, is a positive sense ssRNA virus belonging to the family Coronaviridae. ACE2, a mammalian cell receptor, is used by both SARS and SARS-CoV-2 for cell entry [6,7]. The spike protein of ACE2-binding betacoronaviruses binds to the ACE2 receptor using its receptor binding domain (RBD), resulting in infection of the mammalian cell [8,9]. Standard phylogenetic methods utilizing the RBDs have shown two distinct clades of ACE2-binding RBDs separated by numerous non-ACE2-binding RBDs [10,11,12,13,14,15]. However, by performing hierarchical clustering based on a contextually sensitive composite representation of molecular weight and hydrophobicity of amino acids that comprise the peptide chains, we observed that all known ACE2-binding RBDs cluster within a single well-defined clade [15]. This exposes the possibility of a single ACE2-binding ancestor of the extant ACE2-binding RBDs, which may be shared by all ACE2-binding betacoronaviruses. However, this may also be masked at the sequence level due to the extensive evolution of the RBDs.

While convergent evolution could have resulted in the observed two separate ACE2-binding RBD clades, there is also a possibility of this arising as a result of recombination events between betacoronaviruses and other virus families, a prospect that has been previously explored [16,17]. Nikolaidis et al. reported that such events may be common with the region of the spike gene representing a hotspot. Further, they inferred that recombination has occurred between coronaviruses and toroviruses, reoviruses and astroviruses [17]. Their analyses were primarily based on identifying regions of the genome that present phylogenetic incongruency among varied genomic regions and thus they concluded that these are likely indicative of recombination events between distant viral taxa. Such analyses rely on the sequence alignment of the genomic regions and are, therefore, necessarily bound by the limits of substitution saturation. The method presented by Dixson and Azad [15], based on shared MWHP physicochemical profiles as evaluated using dynamic time warping (hereafter referred to as MWHP PCDTW), can reach beyond saturation (i.e., into the twilight zone of sequence identity) by exposing conservation of physicochemical properties rather than amino acid identity. Further, the method has also been shown to correlate well with structural comparisons of both simulated and experimentally determined structures, thus demonstrating its usefulness in the inference of structural/functional similarity in lieu of homology/de novo modeling or solving of experimental structures [18]. This approach can thus uncover distant homology based strictly on conserved MWHP physicochemical profiles while preserving the contextual basis encoded in the primary sequence; however, in some instances, the effects of convergent evolution cannot be ruled out. Note that even standard alignment-based techniques are also susceptible to the confounding effects of molecular-level convergence [19]. Those effects are generally less pronounced at greater levels of sequence identity but become increasingly pronounced with decreasing identity, particularly in the Twilight Zone of <30% identity. It is the potential functional/structural similarity among RBDs or RBD-like sequences in varied families of viruses that we explore herein by reaching into the Twilight Zone through MWHP physicochemical comparisons.

As of this writing, the COVID-19 pandemic caused by the betacoronavirus SARS-CoV-2 has resulted in the deaths of ~7 million people worldwide [20]. The global medical response brought on by the pandemic has waned, and it appears that a far less deadly endemic derivative of the original zoonotic variant persists. Despite this, the supposed end of the COVID-19 pandemic is not the end of the betacoronavirus concern, instead and as others have stressed, it may be just the beginning [17] since betacoronavirus zoonoses are an enduring problem. The urgency in determining where the progenitor of all three deadly coronaviruses, including SARS-CoV-2, originated and how they acquired the ability to efficiently infect human cells is a paramount concern. Whether or not that search should focus strictly on betacoronaviruses or extend beyond is explored herein. Such a determination will provide valuable insights into the process of evolution that resulted in the highly impactful COVID-19 pandemic and may also enable reliable inferences of when and where another deadly betacoronavirus might arise. That is especially urgent considering that all three of the known deadly human betacoronaviruses have arisen in the past ~20 years, with the most recent, SARS-CoV-2, having a death rate far lower than the death rates of either SARS (~9%) or MERS (~34%) [21]. At any time, a new coronavirus zoonosis could occur whereby a virus that stochastically combines the high infectivity of SARS-CoV-2 [22] and the high death rate of MERS or SARS, may emerge [17]. If/when such an event occurs, then we as a global society will have little technological defense since just a short time of the spread of such a virus would likely pale the mortality that we have seen with SARS-CoV-2, rendering vaccine production and other mitigation efforts, even if they are as rapid as they were with SARS-CoV-2, futile at best. This underscores the need for prediction and prevention methods with regards to coronavirus and, more specifically, betacoronavirus zoonoses. It also underscores the need for vaccines that are of much wider spectrum than the currently administered COVID-19 vaccines, most of which only target the spike protein [17,23].

While recombination between distant viral lineages was once viewed as anomalous and rare, it is now recognized as a major source of variation among viruses [24] including SARS-CoV-2 and more broadly the Sarbecoviruses [24,25]. Positive sense ssRNA viruses, like coronaviruses, are known to experience higher levels of recombination than negative sense ssRNA viruses [24]. It has long been known that coronaviruses experience frequent recombination events within the family Coronaviridae [26,27,28] leading to the genesis of new viruses and it has also been proposed that the ability to bind to human ACE2 receptors, which appears to be key to pathogenicity with regards to SARS-like coronaviruses, is the result of a heretofore unknown recombination event [25,29,30]. Additionally, inter-family recombination among viruses has been shown to occur frequently among some plant viruses [31,32] and several recombination events have been proposed and/or are now accepted to have occurred in mammalian coronaviruses [16,17] sometimes resulting in increased host tropism [32]. Recombination with coronaviruses is also not restricted to Coronaviridae, ssRNA viruses, or just homologous recombination. Inter-family non-homologous recombination has been documented between a coronavirus and a dsRNA bat orthoreovirus [16] and several similar events between coronaviruses and other taxa were also more recently proposed [17], raising the possibility that the apparent uptick in the rate of deadly coronavirus zoonoses that use the ACE2 receptor for human infection could be the result of a heretofore undetected inter-family recombination event that occurred 20+ years ago. While convergent evolution is a possibility, rapid evolution following recombination is a more plausible scenario, which may obfuscate the conservation signals exploited by sequence alignment-based phylogenetic methods. This may help explain the apparent explosion of deadly ACE2-utilizing coronaviruses in the human population over the past 20 years when, prior to 2002, none were known to exist. This also necessitates the development of more robust approaches for discerning such evolutionary scenarios.

In order to explore potentially shared genetic information as the source of ACE2 binding in betacoronaviruses, we analyzed four families of viruses either known to have recombined with coronaviruses or closely related to coronaviruses. The order Nidovirales includes four families; Arteriviridae, Coronaviridae, Mesoniviridae and Roniviridae. Of those, only the Arteriviridae and Cornonaviridae are known to infect mammals [33,34]. Due to the taxonomic proximity to coronaviruses and the fact that they infect mammalian cells, we included arteriviruses in our analyses despite a lack of previously published evidence for recombination between a coronavirus and an arterivirus. The remaining three families analyzed herein, namely astroviruses, reoviruses and toroviruses, were chosen based on the fact that there is documented evidence that each family has experienced recombination with coronaviruses [16,17]. It is worth noting that the two genera of reoviruses included in our study, orthoreoviruses and rotaviruses, are widespread pathogenic mammalian viruses. Additionally, both orthoreoviruses and rotaviruses potentially experience elevated levels of recombination with coronaviruses in coinfected bats and other mammals, including humans, since both are known to circulate to varying degrees in those populations [35,36].

While we do not attempt to propose individual recombination events using MWHP PCDTW, we do hypothesize that unusually high similarity can be observed for RBD sequences across divergent taxa that could arise through the sharing of genetic material through recombination (horizontal transfer), convergence, or vertical descent but loss in multiple lineages. In an effort to assess functional/structural similarity as indicated by MWHP similarity [18] of RBDs and RBD-like sequences between betacoronaviruses and the aforementioned viruses, within four divergent virus families, we performed a hierarchical clustering analysis using MWHP PCDTW [15,18]. Some sequences from the viral families Arteriviridae, Astroviridae and Reoviridae (both from the genera rotavirus and orthoreovirus considered separately) were found to have unusually high MWHP PCDTW similarity to the coronavirus RBDs in regions with similar function to the spike protein of betacoronaviruses, potentially indicating horizontal acquisition followed by divergence. This was especially true for Arteriviridae, in which three glycoprotein 2 (GP2) sequences clustered more closely with RBDs of members of the ACE2-binding betacoronavirus clade than to members of their own virus family.

## 2. Materials and Methods

### 2.1. Waveform-Based Hierarchical Clustering

In order to explore the sharing of ACE2 binding trait among betacoronaviruses and viruses from other families, we analyzed proteins from four virus families using MWHP PCDTW, the technique proposed by Dixson and Azad [15] and further validated using verified structural data found in the SCOP database [18]. The method detects content and contextual MWHP conservation among the amino acid sequences. While it has been described in detail in our previous publications [15,18], we briefly summarize this technique here. MWHP PCDTW defines a scalar for each amino acid in a polypeptide chain. Those scalar values (Table 1) represent a combination of the physicochemical properties of molecular weight and hydrophobicity for each amino acid in the chain. The vector consisting of those scalar values is then converted to a context-aware vector using Equation (1). The context-aware vectors can be visualized as waves of periodically variant amplitude by plotting them in two dimensions where the X-axis represents the amino acid position, and the Y-axis represents the amplitude of each element of the vector at the respective position. For this reason, these vectors are referred to as waveforms herein. The context-aware vectors representing amino acid sequences are then compared in a pairwise fashion using dynamic time warping (DTW), a technique that uses dynamic programming to achieve the optimal alignment of vectors and scores that alignment as the Euclidean distance between optimally aligned vectors [37]. Following DTW, those Euclidean distances can be used in downstream analyses such as the hierarchical clustering performed herein. The families studied here using this method included Arteriviridae, Astroviridae, Reoviridae (both from the genera rotavirus and orthoreovirus considered separately) and Toroviridae, and the distance networks were presented in dendrogram (bifurcating tree) form.

The arterivirus sequences included in our analyses were separated according to individual surface glycoproteins, and separate dendrograms were produced for each of those proteins. Those included the surface glycoproteins 2–5 (GP2–GP5). For the other virus families, the surface proteins responsible for host cell entry were analyzed. In all cases, the receptor binding domain of SARS-CoV-2 (wild type UNIPROT Acc. Num.: P0DTC2) was used as the query sequence to identify regions of similarity in the proteins of other viruses. Following identification of the region of highest similarity to the RBD of SARS-CoV-2 using DTW, a dataset containing only those regions and 51 betacoronavirus RBDs, including the SARS-CoV-2 RBD was subjected to waveform-based UPGMA [38] and neighbor-joining (NJ) [39] hierarchical clustering [15]. The congruency of UPGMA and NJ dendrograms was determined using common nodes and inverse, normalized Robinson-Foulds metrics as calculated using the dendextend [40] and treedist [41] packages in R. Of the 51 betacoronavirus RBDs considered, 16 are known to or have been bioinformatically predicted to bind to ACE2 and the rest are neither known nor predicted to bind to ACE2 [6,7,8,15,42,43,44,45,46,47,48]. The full list of sequences, as well as how they were grouped for each analysis, can be found in File S1 of Supplementary Materials. Following the initial comparisons, a follow-up UPGMA dendrogram was produced using the same methodology previously described and by combining the arterivirus, torovirus and betacoronavirus datasets. That dendrogram was produced to better resolve notable findings in the dendrograms from the initial analysis. Additionally, an unrooted dendrogram was produced to collectively visualize the comparisons of astroviruses and orthoreoviruses to betacoronaviruses. All dendrograms were prepared for publication using iTOL at https://itol.embl.de (accessed 4 October 2024) [49]. The Python script used is available at https://github.com/JamberFX/MWHP_Subsequence_Match_Clustering (accessed 4 October 2024).

(1) $$Y=\frac{NoteVec\left[l\right]+\left(\frac{NoteVec\left[l\right]+NoteVec\left[l+1\right]}{2}\right)+\left(\frac{NoteVec\left[l\right]+NoteVec\left[l-1\right]}{2}\right)}{2}$$

where

*Y* = *Final Vector Element*

*NoteVec* = *Amino Acid Scalar Value*

### 2.2. Pairwise Global Alignment

Following waveform-based hierarchical clustering, only three non-coronavirus sequences were found to cluster exclusively with ACE2-binding betacoronavirus sequences in both UPGMA and NJ analyses. Those were all arterivirus GP2 sequences. Those three sequences were placed in a separate dataset with the 51 betacoronavirus RBD sequences that were then subjected to pairwise global alignment with the Bio.Align module of BioPython [50], using BLOSUM45 substitution matrix, gap opening penalty of −3 and gap extension penalty of −1. Pairwise alignment identity percentages were compiled into a matrix that was used to make a heatmap using the MatplotLib [51] and Seaborn [52] Python packages. Of note, NJ dendrograms were not subjected to similar analyses because the NJ dendrograms showed inconsistencies whereby non-ACE2-binding betacoronavirus sequences clustered within the ACE2-binding clade, a non-parsimonious result that was not only absent in the UPGMA dendrograms but could also indicate that NJ clustering is inappropriate for use on MWHP physicochemical vectors for reasons that are beyond the scope of this study.

### 2.3. In Silico Structural Design

Despite the recent publication of a validation study that illuminated a strong relationship between MWHP PCDTW distances and structural/functional divergences for a large number of sequences in the verified SCOP database [18], it was deemed necessary to show here a similar relationship regarding betacoronavirus RBDs. To demonstrate the relationship between the MWHP physicochemical properties of an amino acid sequence and the three-dimensional structure possible for that polypeptide and reinforce previous functional validation of MWHP PCDTW [15], we designed a fully divergent version of the 6M0J SARS-CoV-2 RBD from the protein data bank at https://www.rcsb.org/ (accessed 3 April 2024). This was accomplished by using the amino acid scalar values presented previously by Dixson and Azad (Table 1) to find the least different amino acids at each residue position [15]. That sequence was then subjected to homology modeling on the Swiss-Model server using the structure for 6M0J as the template. The resulting structure was compared to 6M0J in terms of RMSD and TM-Score using Chimera X and the TM-Align server at https://zhanggroup.org/TM-align/ (accessed 3 April 2024) [53,54].

## 3. Results

**Figure 1:**
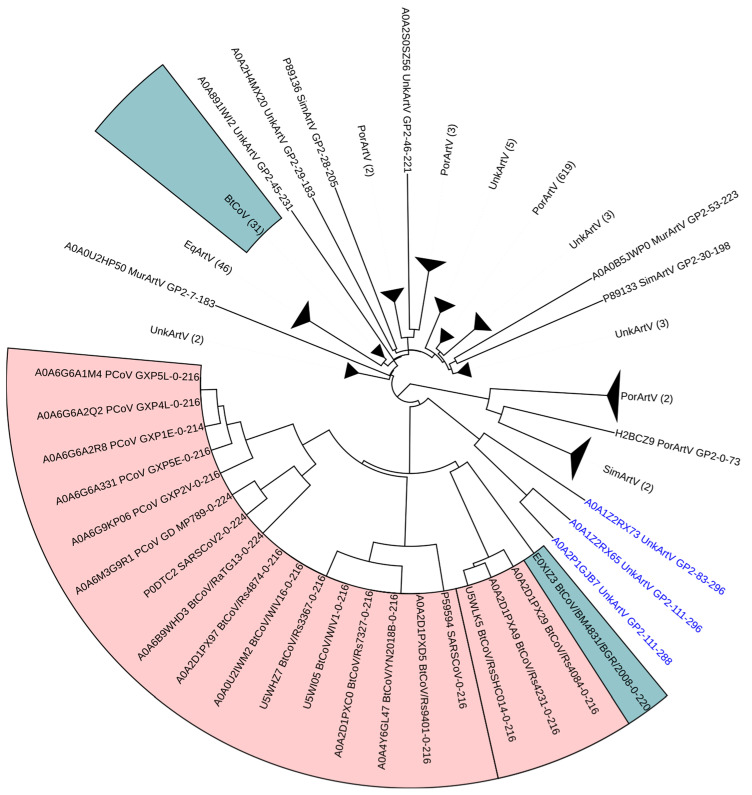
Dendrogram of betacoronavirus spike protein RBD sequences and arterivirus GP2 sequences constructed using MWHP PCDTW with Euclidean distance and UPGMA hierarchical clustering. ACE2-binding betacoronavirus sequences are highlighted in red, and non-ACE2-binding betacoronavirus sequences are highlighted in blue. In most cases, the host organism is encoded into the taxa label. In some cases, the host was labeled as “UnkArtV.” In those cases, the Uniprot record should be consulted for additional information concerning the host organism. The three arterivirus sequences that cluster near the ACE2-binding betacoronaviruses have blue text labels and are from Oliver’s Shrew. All black text labels not in a colored box represent Arterivirus GP2 sequences that did not cluster closely with the betacoronavirus sequences.

An MWHP physicochemical waveform-based UPGMA hierarchical clustering analysis of sequences from 4 virus families obtained by comparison to the RBD of the ACE2-binding betacoronavirus, SARS-CoV-2, as the query, revealed that the viral families Arteriviridae, Astroviridae, Reoviridae (both rotaviruses and orthoreoviruses considered separately) and Toroviridae have unusually high similarity to betacoronaviruses in regions similar to the RBD of betacoronaviruses, potentially indicating a sharing of genetic information by distant viral lineages (Figure 1 and Supplementary Figures S1–S7). NJ dendrograms for the same datasets were considered less reliable since the clades formed did not accurately depict known functional classifications regarding the ability to bind to the ACE2 receptor. Despite this, they were included herein for thoroughness (Supplementary Figures S8–S14). Henceforth, reference is made exclusively to the UPGMA dendrograms unless explicitly stated.

Each of the four arterivirus surface glycoproteins (GP2-GP5) was analyzed separately. The comparison between arterivirus GP2 sequences and betacoronavirus RBD sequences depicted in Figure 1 revealed that three arterivirus GP2 sequences clustered relatively closely with RBDs of ACE2-binding betacoronaviruses, a finding that in the context of our analyses is unique to the arterivirus GP2 comparison. The majority of non-ACE2-binding betacoronavirus sequences clustered with arterivirus GP2 sequences that were isolated from equids, and three arterivirus GP2 sequences isolated from rodents were found between the clade containing the ACE2-binding betacoronaviruses and the clade containing the majority of non-ACE2-binding betacoronaviruses. The unusual placement of three arterivirus GP2 sequences within a clade with RBDs of ACE2-binding betacoronaviruses suggests a potential ancient horizontal genetic exchange between betacoronaviruses and arteriviruses. The cladistic pattern displayed in Figure 1 suggests a potential acquisition of the betacoronavirus RBD sequence by arterivirus(es) and divergence since the acquisition. In contrast, the arterivirus GP3-GP5 sequences showed no intermingling of betacoronavirus clade sequences with arterivirus sequences. These analyses and the resulting dendrograms (Supplementary Figures S1–S3) provide a baseline for the remaining topologies presented herein that, to varying degrees, show topological evidence of intermingled evolutionary associations.

The astrovirus capsid polyprotein sequences, when compared to betacoronavirus RBD sequences, present a topology, as depicted in Supplementary Figure S4, whereby ACE2-binding betacoronaviruses are in a single clade, which has a sister clade containing four non-ACE2-binding betacoronaviruses. The larger clade containing those two clades is a sister clade to a clade containing three sub-clades. The first of those sub-clades represents bovine astroviruses, the second sub-clade represents 31 non-ACE2-binding betacoronaviruses, several fish astroviruses and a single human astrovirus, and the third sub-clade represents a single macaque astrovirus. Similar to the arterivirus GP2 comparison, this topology illuminates the possibility of ancient recombination events between these viral lineages, potentially between ancestors of extant betacoronaviruses and astroviruses. However, the topology of astrovirus and betacoronavirus sequences was not nearly as strong of an indicator of this as was observed for the arterivirus GP2 sequences.

Two genera of reoviruses were considered separately. Those were the rotaviruses and the orthoreoviruses. Three rotavirus sequences appear to cluster very closely with ACE2-binding betacoronavirus RBD sequences in Supplementary Figure S5. However, upon closer examination of the database records, those sequences were found to be synthetic chimeric constructs consisting of rotavirus and betacoronavirus sequences. The remaining topology shows a bifurcation of the ACE2-binding and non-ACE2-binding betacoronaviruses by rotaviruses. The orthoreovirus analysis depicted in Supplementary Figure S6 shows a similar topological pattern as seen in the aforementioned comparisons where the betacoronavirus sequences are split into two clades, one containing primarily ACE2-binding betacoronavirus sequences and the other with a single cohesive clade of non-ACE2-binding betacoronavirus sequences, separated by several orthoreovirus sequences. Much like with the other families considered, this may be indicative of ancient recombination events involving ancestors of orthoreoviruses and betacoronaviruses with respect to the RBD. This may also indicate two separate modes of evolution resulting in each of the clades containing the non-ACE2-binding and ACE2-binding betacoronaviruses. Despite this, and much like the astrovirus/betacoronavirus topology, the inferred topology was not nearly as strong of an indicator of potential ancient recombination as was observed for the arterivirus GP2 sequences. To better visualize the observed pattern with non-betacoronavirus sequences placed between ACE2-binding and non-ACE2-binding betacoronaviruses sequences, we produced an unrooted dendrogram depicting relationships, assessed in terms of MWHP PCDTW similarity among betacoronaviruses, astroviruses and orthoreoviruses sequences (Figure S15). The overall star-like pattern of that dendrogram suggests a common ancestor of the betacoronavirus, astrovirus and orthoreovirus sequences, while the fact that a subset of both astroviruses and orthoreoviruses cluster more closely with non-ACE2-binding betacoronaviruses than they do with members of their respective taxa supports the supposition of recombination between ancestors of non-ACE2-binding betacoronaviruses and some astroviruses and orthoreoviruses.

The comparison of betacoronavirus RBDs to torovirus RBDs, as shown in Supplementary Figure S7, resulted in a topology that was unique with respect to the other comparisons made herein in that not only were the betacoronaviruses split into two clades, as in the other comparisons, the clade containing only non-ACE2-binding betacoronaviruses was further bifurcated by 30 toroviruses. This is indicative of a likely ancient horizontal transfer of a non-ACE2-binding betacoronavirus sequence to the common ancestor of these 30 toroviruses.

**Figure 2:**
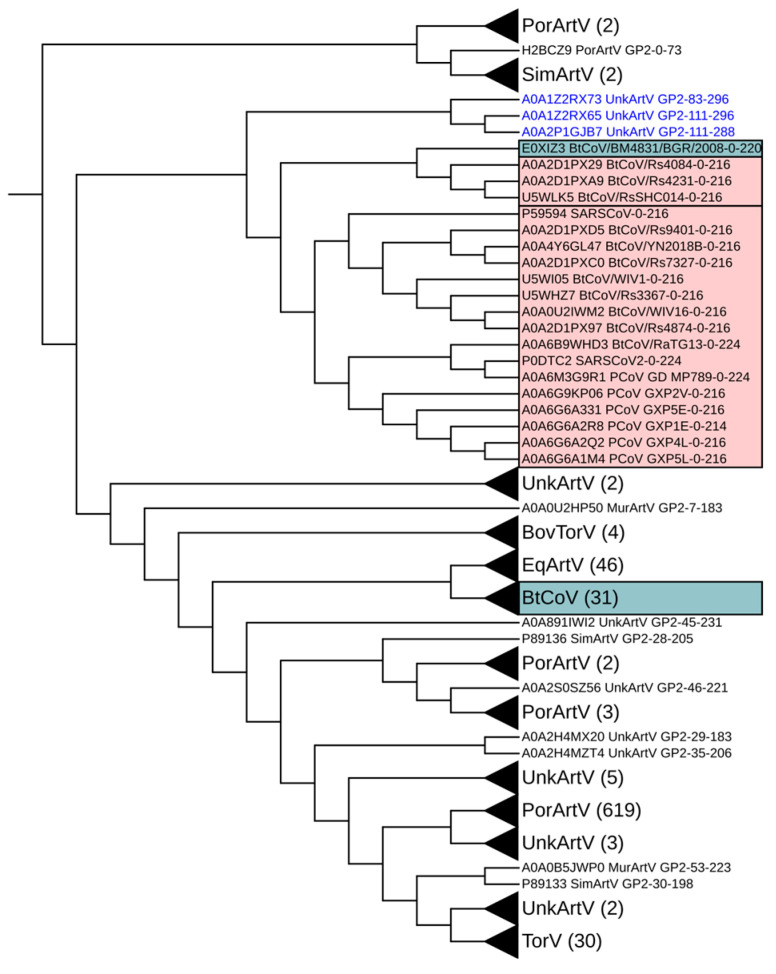
Dendrogram of betacoronavirus spike protein RBD sequences, arterivirus GP2 sequences and torovirus sequences constructed using MWHP PCDTW with Euclidean distance and UPGMA hierarchical clustering. ACE2-binding betacoronavirus sequences are highlighted in red, and non-ACE2-binding betacoronavirus sequences are highlighted in blue. Three Arterivirus GP2 sequences that are unique within this study, in that they cluster very closely with ACE2-binding betacoronavirus sequence, are labeled with blue text. This combined dendrogram underscores the similarity of the three arterivirus GP2 sequences to those of the ACE2-binding betacoronavirus sequences.

In order to further explore the notable relationships mentioned above for the betacoronavirus/arterivirus GP2 sequences and the betacoronavirus/torovirus sequences, we repeated our MWHP PCDTW UPGMA analysis using a combined dataset, including the betacoronavirus, arterivirus GP2 and torovirus sequences (Figure 2).The topology of that dendrogram highlights closer proximity among betacoronavirus/arterivirus GP2 sequences than among betacoronavirus/torovirus sequences, as assessed in terms of MWHP PCDTW similarity. The dendrogram depicts several nodes leading to intermingled combinations of sequences from each of the three families, potentially indicating multiple past recombination events or convergent evolution. Of note is that the ACE2-binding and non-ACE2-binding betacoronavirus sequences have remained in cohesive clades, respectively, across all comparisons made.

**Figure 3:**
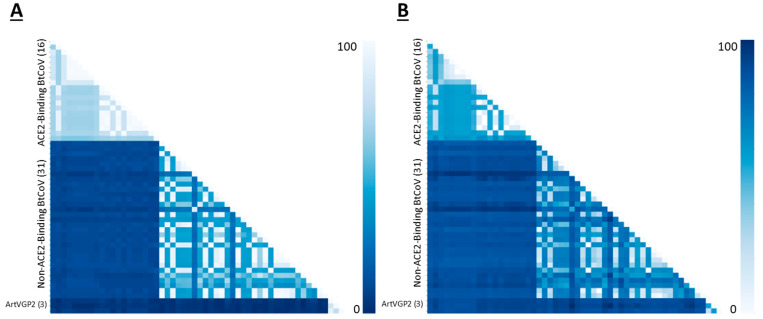
(**A**) Pairwise global alignment identity matrix showing identities for all pairwise comparisons of 51 betacoronavirus spike protein RBD sequences and three arterivirus GP2 sequences. (**B**) Pairwise distance matrix showing MWHP PCDTW distances which have been scaled to 100 for all pairwise comparisons of 51 betacoronavirus spike protein RBD sequences and three arterivirus GP2 (ArtVGP2) sequences. The Pearson Correlation Coefficient for the values in A and B is 0.44 with a *p*-Value of 4.98 × 10^−140^.

**Figure 4:**
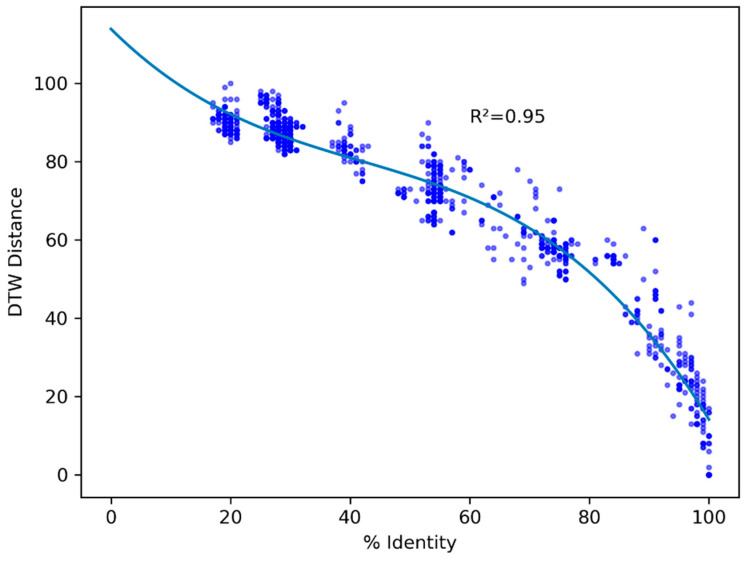
Scatterplot of pairwise global alignment identity percentages and MWHP PCDTW distances scaled to 100 for all comparisons made between arterivirus GP2 sequences and betacoronavirus sequences underlying the dendrogram shown in Figure 1. The line shown is a polynomial regression line. This shows that, in general terms, the two signals are not different ways of measuring the same signal. In other words, there is additional information in the MWHP PCDTW signal that is not in the identities.

To explore the relationships between RBDs of ACE2-binding betacoronaviruses and the three arterivirus GP2 sequences which cluster with those ACE2-binding sequences (see Figure 1 and Supplemental Figure S8), we performed a pairwise global alignment comparing those three sequences to the betacoronavirus sequences included in our MWHP PCDTW analyses. Pairwise alignment identity percentages between ACE2-binding betacoronaviruses and the arterivirus GP2 sequences ranged from 22–26% (Figure 3), confirming that these relationships fall well into the twilight zone of <30% protein sequence identity where standard alignment-based techniques cannot distinguish reliably between this level of similarity arising due to homology and due to random chance [55,56]. To determine the nature of the relationship between the PCDTW signal and that of the pairwise alignment, we calculated the Pearson Correlation Coefficient and *p*-Value for the two datasets. There was a moderate positive correlation between the two variables, with a correlation coefficient of 0.44 and a *p*-Value of 4.98 × 10^-140^. Further, to visually evaluate the nature of the MWHP PCDTW signal, we plotted the pairwise identities against the MWHP PCDTW distances that were scaled to 100. This comparison, which is illustrated in Figure 4, demonstrates that although the two signals are correlated, they are different signals and additional information is contained in the MWHP PCDTW signal. This reaffirms the validity of using the MWHP PCDTW signal to make comparisons of distantly related sequences.

**Figure 5:**
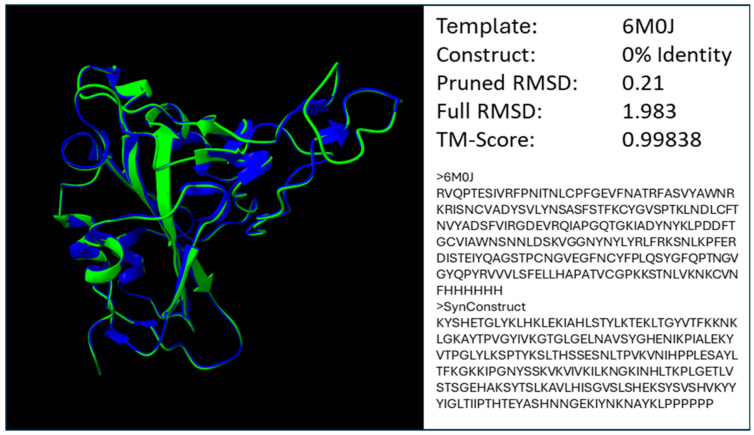
Superimposition of the SARS-CoV-2 RBD (6MOJ) and a synthetic construct homology model which has 0% identity to 6MOJ. The synthetic model exhibits an extremely low pruned RMSD value of 0.21 (~96% of residues considered) and also a very low TM-Score indicating that the two structures are nearly identical [15,18].

To demonstrate that the molecular weight and hydrophobicity physicochemical properties used in MWHP PCDTW are closely related to the observed structure of the RBD and therefore validate the use of MWHP PCDTW distances in relational studies beyond the functional validations provided by Dixson and Azad [15,18], we created a fully divergent (0% identity) SARS-CoV-2 RBD in silico construct that, as can be seen in Figure 5, is extremely similar in structure to the wildtype RBD with a pruned RMSD value of 0.21 (96% of the available residue pairs considered), a full RMSD value of 1.983 and a TM-Score of 0.99838.

## 4. Discussion

UPGMA and NJ hierarchical clustering were used to cluster distance matrices derived from vectors of transformed amino acid sequences, which, in a content and context- aware fashion, represent the MWHP physicochemical properties of those sequences. The transformation to MWHP physicochemical vectors, in effect, removes the actual sequence from any similarity measure derived from those vectors. This in turn has the effect of masking both homoplasy and the clock-like nature of evolution, affecting standard sequence-based analyses. While the removal of those two factors may have consequences with regard to the available standard techniques to analyze a group of sequences, it also allows for the use of our method (MWHP PCDTW) [15,18] that emphasizes functional change and minimizes the effects of silent and homoplastic evolution. This is perhaps the reason why the simpler UPGMA clustering technique results in the clustering of our vectors that are in agreement with known functional classification, and NJ proves less reliable in that respect both herein and previously [15]. Further, the validity of using MWHP PCDTW to evaluate structural/functional relatedness with respect to the betacoronavirus spike RBD is established by the functional classification of ACE2-binding RBDs resolved by Dixson and Azad [15], notable correlation of MWHP PCDTW distances with structural divergences among protein domains in the SCOP protein domain family database [18], as well as the structural agreement demonstrated by in silico modeling as shown in Figure 5. The synthetic structure designed to resemble the SARS-CoV-2 RBD and depicted in Figure 5 is notable because it is nearly identical to the wild type SARS-CoV-2 RBD in terms of pruned RMSD as well as TM-Score despite 0% identity, thus validating the use of MWHP vectors both in the design of synthetic constructs and also as representative vectors in distance calculations using MWHP PCDTW.

NJ is a technique that was designed to primarily address the ultrametric requirement of UPGMA [39,57]. In other words, it adjusts for the assumption with UPGMA that all analyzed sequences must be equally divergent from the mid-point root of the tree (i.e., have a constant molecular clock). However, the UPGMA assumption is completely reasonable for datasets representing functional/structural change with limited homoplasy, which is inherent to the MWHP physicochemical vectors used in MWHP PCDTW. Since NJ is a technique which introduced corrections for the ultrametric requirement, it is perhaps those corrections which are not only unneeded when analyzing MWHP PCDTW vectors but may also be contributing to the aberrant results seen in the NJ trees herein and in previous applications of MWHP PCDTW (Supplementary Figures S8–S14) [15]. For these reasons, we suggest that, henceforth, UPGMA be used as the primary means of clustering for MWHP PCDTW-derived distance matrices.

Unusually high similarity in sequences from among otherwise distant taxa can be indicative of recombination (horizontal exchange) or convergent evolution. In data that correlates well with functional/structural evolution, such as MWHP PCDTW distances [18], it may be impossible to rule out convergence when sequences with low amino acid identity also appear to be very similar based on MWHP PCDTW distance assessment. While recombination is not specifically addressed, evidence supports the supposition that recombination could have played a role in the acquisition of ACE2 binding within betacoronaviruses [7,25,29]. We have shown in most of the virus families analyzed that the betacoronavirus clade includes some non-coronavirus sequences, exposing the possibility that while convergence cannot be ruled out, those sequences likely could have been mobilized from betacoronaviruses to other viral lineages through horizontal transfer, potentially via recombination.

The overall UPGMA topology presented for each separate virus family as compared to betacoronaviruses was similar among all comparisons except for the arterivirus GP3-GP5 topologies (Supplementary Figures S1–S3) that show no intermingling of betacoronavirus and arterivirus sequences. Such conservation of topology is likely indicative of structural, and, thus, functional conservation that may be difficult to discern using standard alignment-based techniques, specifically when that conservation is within the twilight zone of sequence identity [55,58]. The sequences used herein are within the twilight zone, as shown in Figure 3A, and yet MWHP PCDTW inferred structural/functional similarity has been maintained across divergent taxa, most likely via purifying selection for the underlying structure/function (see Figure 1 and Figure 2). By inferring structural similarity between some betacoronavirus sequences and functionally similar sequences in other taxa, we have exposed potential evidence for ancient horizontal transfer of betacoronaviruses sequences to (or from) other viral lineages and their substantial divergence since the transfer, which are otherwise difficult to discern using standard sequence alignment-based techniques. This is particularly evident in Figure 2, where the high similarity between three arterivirus GP2 sequences and ACE2-binding betacoronavirus sequences maintained cladal cohesion despite the introduction of torovirus sequences, thus underscoring the similarity of these sequences and the robustness of MWHP PCDTW in uncovering potentially ancient recombination events.

The fact that all of our UPGMA dendrograms, with the exception of those containing arterivirus GP3–GP5 sequences (Figure 1 and Supplemental Figures S4–S7), show similar topologies where the clade containing primarily ACE2-binding betacoronaviruses is separated, from the clade containing primarily non-ACE2-binding betacoronaviruses, by sequences within each of the respective diverse virus families, is reflective of similar evolutionary processes that have proceeded separately yet in parallel within each of the virus families considered while at the same time potentially experiencing periodic recombination events. This would be similar to concerted evolution as experienced in eukaryotic multigene families [59].

In most cases, it may almost be impossible to reliably infer homology using primary sequences that have experienced high levels of evolutionary divergence due to saturation at the nucleotide and amino acid levels. Saturation at the amino acid level is related to the similarity of physicochemical or structural characteristics, whereas saturation at the nucleotide level is related to the redundancy of the genetic code. In a functional sense, however, saturation at the amino acid level is related to the redundancy of the physicochemical characteristics among the entire suite of available amino acids. As Lolkema et al. pointed out, each individual amino acid position has specific physicochemical requirements which are entirely unique to that position. However, it is also true that depending on the breadth of physicochemical requirements, more than one amino acid may be able to fulfil those requirements such that, if given enough time, homology may be indiscernible by sequence comparisons alone. Our synthetic construct in Figure 5, which is 0% identical to the SARS-CoV-2 RBD yet is almost indiscernible, structurally supports that proposition. Further, given enough evolutionary time, an alternate amino acid does not have to be the amino acid that is dictated in the genetic code as being the simplest to transition to. This led to Lolkema and Slotboom’s statement that evolution of homologous sequences is tolerant of multiple amino acid substitutions [58]. It is at variance, however, with standard methodology that assumes that over shorter periods of evolutionary time, amino acid substitution is far more restrictive than nucleotide substitution. Our newly proposed technique (MWHP PCDTW) allows one to reach beyond the confines of amino acid substitution saturation by encoding a string of residues as a physicochemical waveform, representing the combined physicochemical properties of molecular weight and hydrophobicity, that can be imperfectly matched to other waveforms and, thus, may allow for similarity detection over greater periods of evolutionary time. Herein, this has allowed us to identify three potentially ancient xenologs to the ACE2-binding betacoronavirus spike protein RBD domain in the family Arteriviridae. Further application of this technique across additional taxa may allow for the inference of homologs, both recent and ancient, and specifically the xenologs, of the ACE2-binding betacoronavirus RBDs.

In conclusion, we reiterate that a content/context physicochemical vector such as the MWHP vector used herein can be used to decipher shared structure/function as well as infer deep evolutionary relationships [18]. Previous studies have highlighted the limitations of standard alignment-based phylogenetic techniques, such as their inability to discriminate between equally probable topologies [60]. There is an unmet need to develop alternative strategies. MWHP PCDTW is a step forward in this direction. MWHP PCDTW has the ability to unveil structural characteristics and, thus, the biological significance embedded within topological inferences. MWHP PCDTW complements the standard techniques that are robust in phylogenetic inference when sequence identity is moderate to high, but their capability degrades at lower levels of identity [19,61]. Our study shines a new light on inferred structural/functional conservation of ACE2-binding betacoronavirus RBDs and their potential mobilization across viral lineages, which warrants further investigation as their ability to bind to ACE2 may be a trait shared by multiple viral lineages.

## Figures and Tables

**Table 1:** Note values (NoteVec) for each amino acid representing a combination of molecular weight and hydrophobicity as described previously [15,18]. These values are assigned to respective amino acids of a polypeptide and converted into a vector for further analysis using Equation (1).

AA	Value	AA	Value	AA	Value
G	0.3064	Q	−6.8806	Y	42.9375
A	15.6497	E	5.547	H	−22.8297
L	60.7668	S	−2.3381	R	−11.7423
M	52.1362	P	−23.9905	N	−17.1576
F	76.6645	V	40.4569	D	−33.9918
W	97.0000	I	60.1592	T	7.0585
K	−15.8315	C	27.1418		

## Data Availability

The sequences used in this study are provided in File S1 of the Supplementary Materials. The Python script used for PCDTW analysis is available at https://github.com/JamberFX/MWHP_Subsequence_Match_Clustering, accessed 3 April 2024.

## Supplementary Materials

The following supporting information can be downloaded at: https://www.mdpi.com/article/10.3390/microorganisms12102021/s1, File S1: Sequences used in all analyses; Figure S1: Dendrogram of betacoronavirus spike protein RBD sequences and arterivirus GP3 sequences con-structed using MWHP PCDTW with Euclidean distance and hierarchical clustering; Figure S2: Dendrogram of betacoronavirus spike protein RBD sequences and arterivirus GP4 sequences con-structed using MWHP PCDTW with Euclidean distance and UPGMA clustering; Figure S3: Dendrogram of betacoronavirus spike protein RBD sequences and arterivirus GP5 sequences con-structed using MWHP PCDTW with Euclidean distance and UPGMA clustering; Figure S4: Dendrogram of betacoronavirus spike protein RBD sequences and astrovirus VP90 sequences constructed using MWHP PCDTW with Euclidean distance and UPGMA clustering; Figure S5: Dendrogram of betacoronavirus spike protein RBD sequences and orthoreovirus S1 sequences constructed using MWHP PCDTW with Euclidean distance and UPGMA clustering; Figure S6: Dendrogram of betacoronavirus spike protein RBD sequences and rotavirus spike protein se-quences constructed using MWHP PCDTW with Euclidean distance and UPGMA clustering, Figure S7: Dendrogram of betacoronavirus spike protein RBD sequences and torovirus spike protein se-quences constructed using MWHP PCDTW with Euclidean distance and UPGMA clustering, Figure S8: Dendrogram of betacoronavirus spike protein RBD sequences and arterivirus GP2 sequences con-structed using MWHP PCDTW with Euclidean distance and neighbor-joining clustering, Figure S9: Dendrogram of betacoronavirus spike protein RBD sequences and arterivirus GP3 sequences con-structed using MWHP PCDTW with Euclidean distance and neighbor-joining clustering, Figure S10: Dendrogram of betacoronavirus spike protein RBD sequences and arterivirus GP4 sequences con-structed using MWHP PCDTW with Euclidean distance and neighbor-joining clustering, Figure S11: Dendrogram of betacoronavirus spike protein RBD sequences and arterivirus GP5 sequences con-structed using MWHP PCDTW with Euclidean distance and neighbor-joining clustering, Figure S12: Dendrogram of betacoronavirus spike protein RBD sequences and astrovirus VP90 sequences constructed using MWHP PCDTW with Euclidean distance and neighbor-joining clustering, Figure S13: Dendrogram of betacoronavirus spike protein RBD sequences and orthoreovirus S1 sequences constructed using MWHP PCDTW with Euclidean distance and neighbor-joining clustering, Figure S14: Dendrogram of betacoronavirus spike protein RBD sequences and torovirus spike protein se-quences constructed using MWHP PCDTW with Euclidean distance and neighbor-joining clustering, Figure S15: Unrooted UPGMA dendrogram depicting the MWHP PCDTW similarity among betacoronaviruses, astroviruses and orthoreoviruses.
