# Supplementary material for: An Analysis of Combined Molecular Weight and Hydrophobicity Similarity between the Amino Acid Sequences of Spike Protein Receptor Binding Domains of Betacoronaviruses and Functionally Similar Sequences from Other Virus Families
Source: Microorganisms. 2024 Oct 5;12(10):2021. doi: 10.3390/microorganisms12102021 (PMC11510113; doi:10.3390/microorganisms12102021)
Supplement: Supplementary file 1 [file microorganisms-12-02021-s001.zip › microorganisms-3213286-Supplementary Figures S1-S15.pdf]

Supplementary Materials

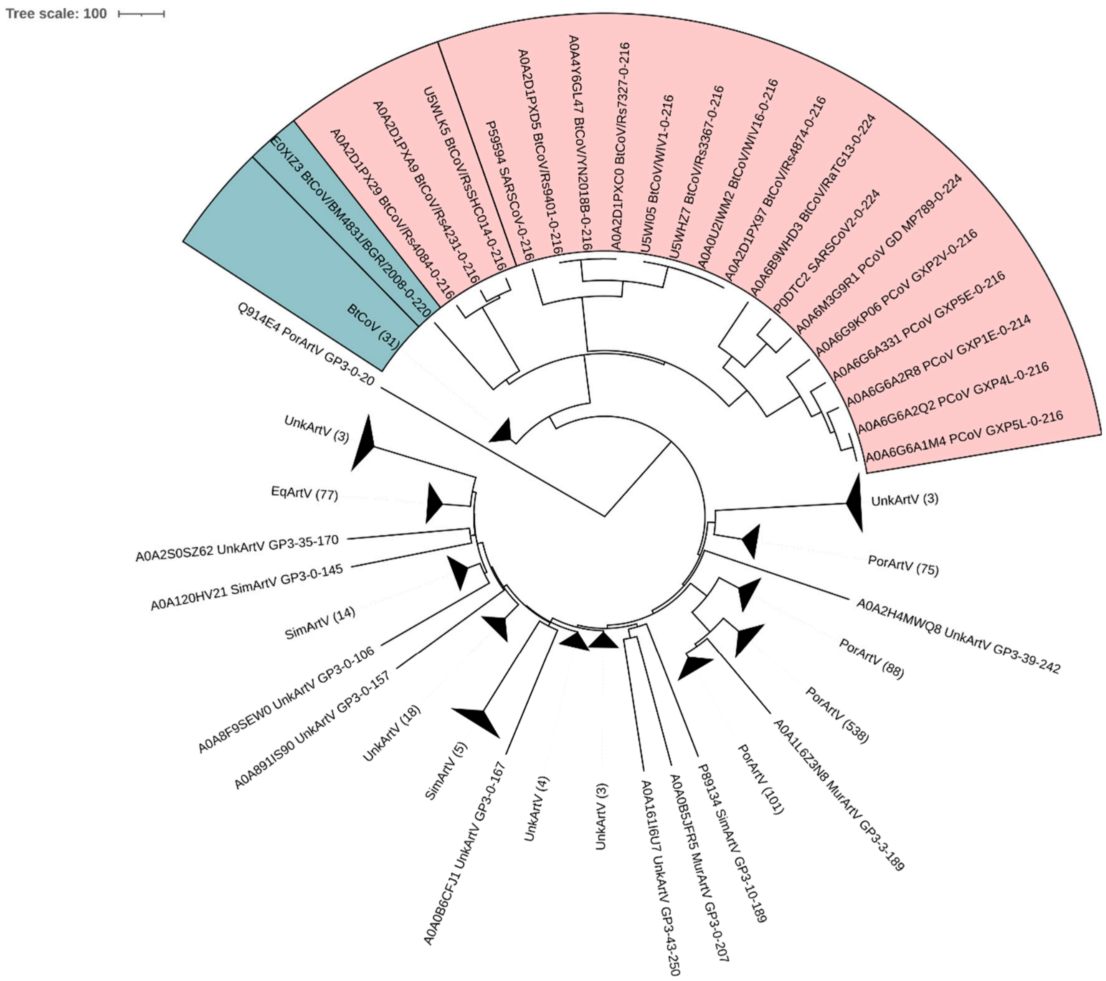

Figure S1: Dendrogram of betacoronavirus spike protein RBD sequences and arterivirus GP3 sequences constructed using MWHP PCDTW with Euclidean distance and hierarchical clustering. ACE2-binding betacoronavirus sequences are in red boxes and non-ACE2-binding betacoronavirus sequences are in blue boxes. Arterivirus sequences are not in boxes.

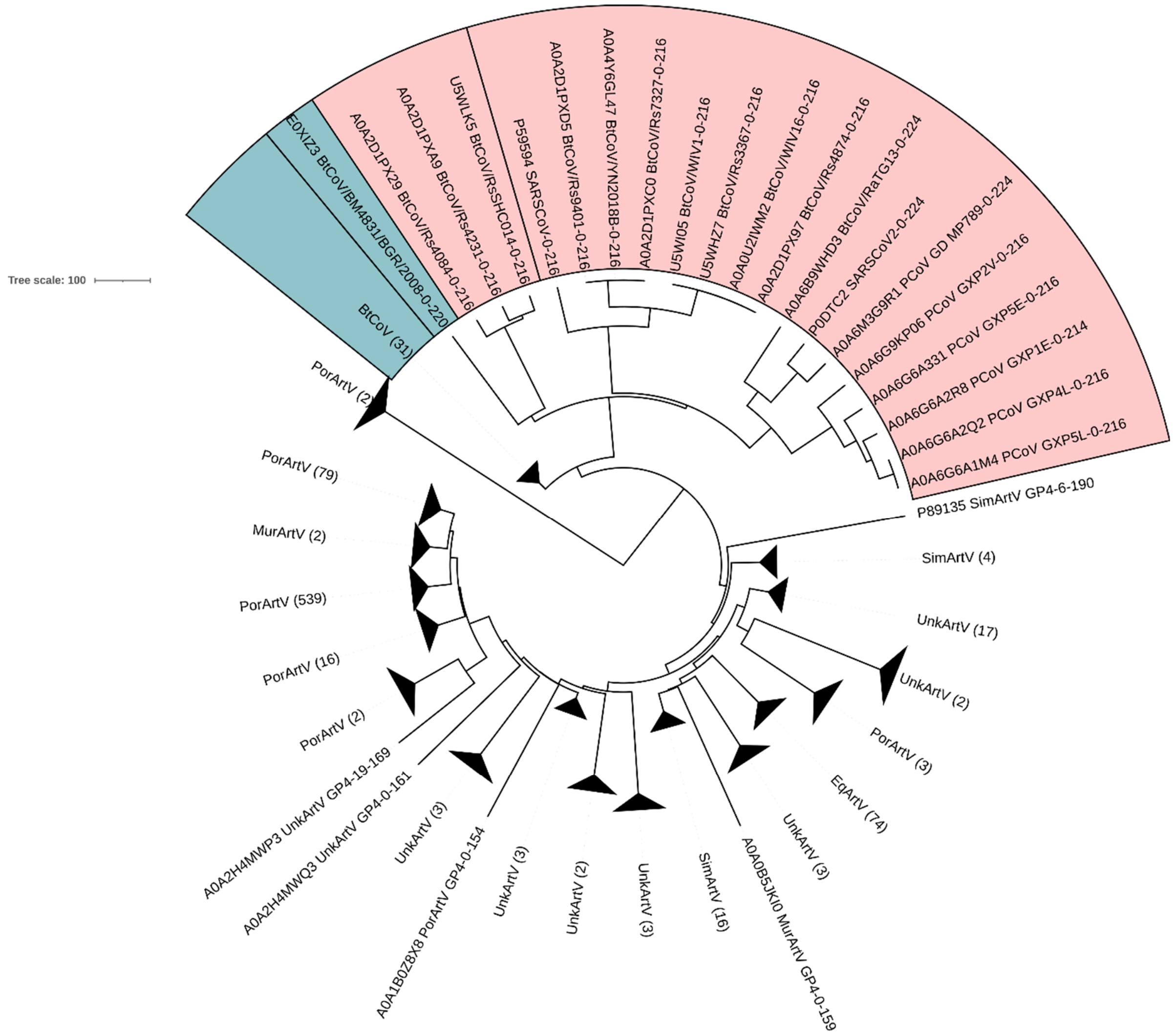

Figure S2: Dendrogram of betacoronavirus spike protein RBD sequences and arterivirus GP4 sequences constructed using MWHP PCDTW with Euclidean distance and UPGMA clustering. ACE2-binding betacoronavirus sequences are in red boxes and non-ACE2-binding betacoronavirus sequences are in blue boxes. Arterivirus sequences are not in boxes.

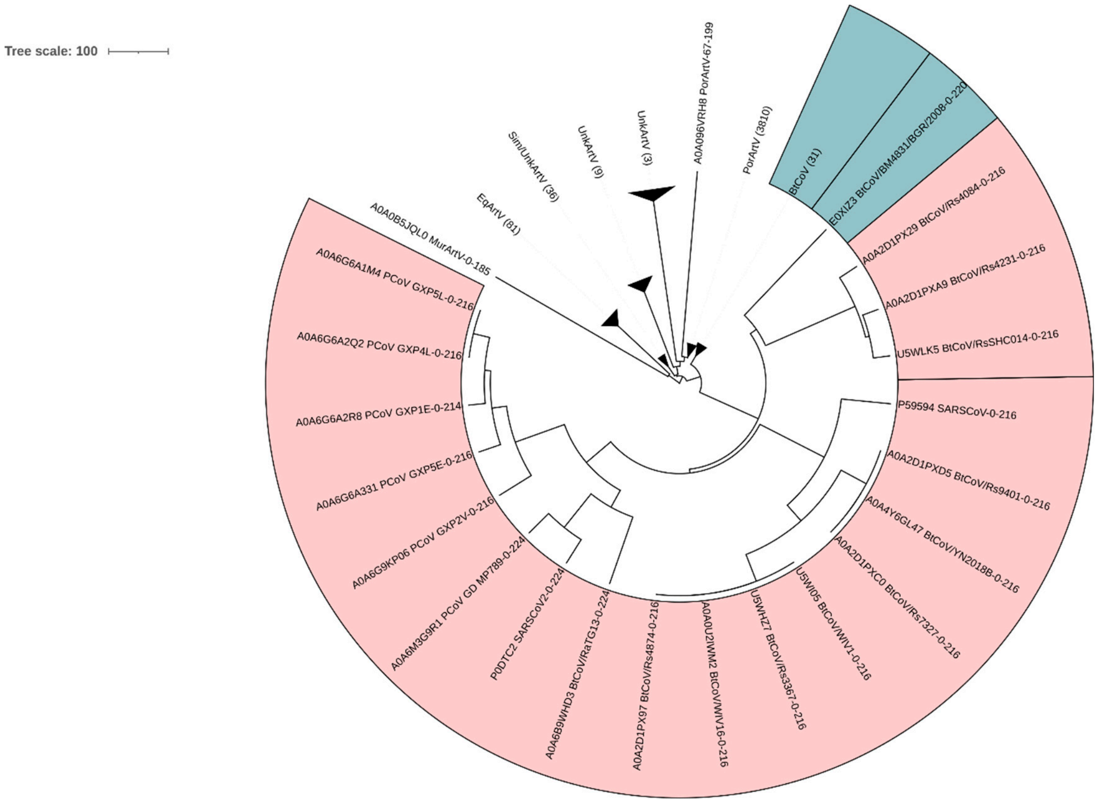

Figure S3: Dendrogram of betacoronavirus spike protein RBD sequences and arterivirus GP5 sequences constructed using MWHP PCDTW with Euclidean distance and UPGMA clustering. ACE2-binding betacoronavirus sequences are in red boxes and non-ACE2-binding betacoronavirus sequences are in blue boxes. Arterivirus sequences are not in boxes.

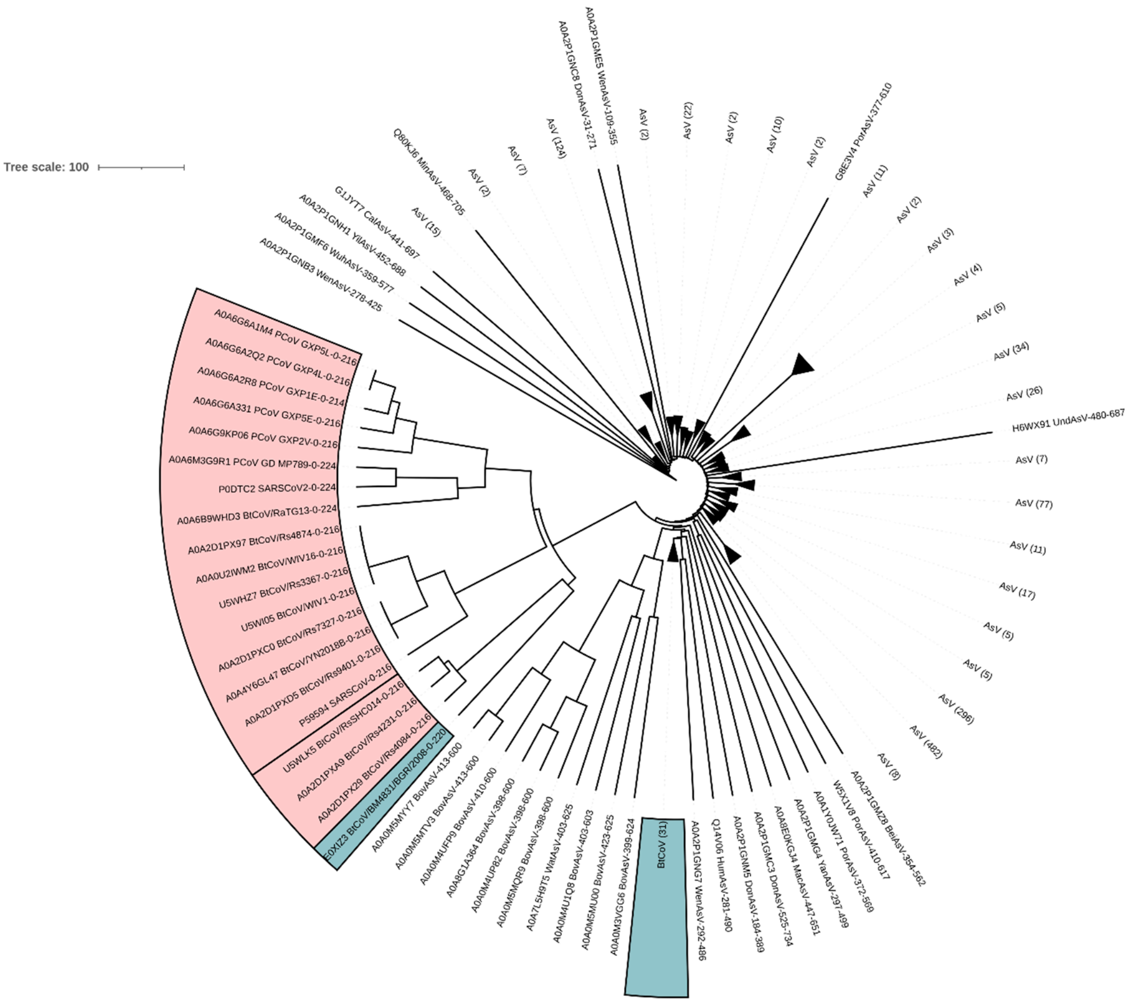

Figure S4: Dendrogram of betacoronavirus spike protein RBD sequences and astrovirus VP90 sequences constructed using MWHP PCDTW with Euclidean distance and UPGMA clustering. ACE2-binding betacoronavirus sequences are in red boxes and non-ACE2-binding betacoronavirus sequences are in blue boxes. Astrovirus sequences are not in boxes.

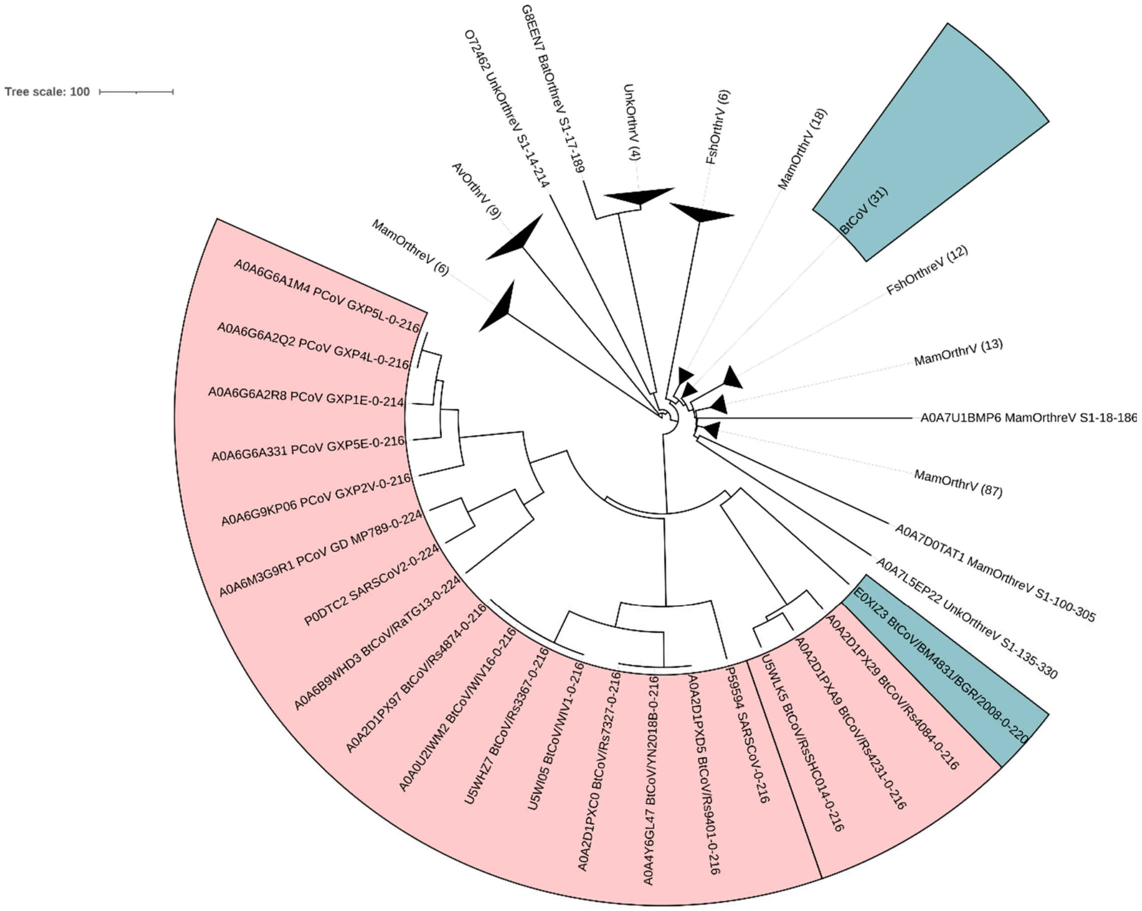

Figure S5: Dendrogram of betacoronavirus spike protein RBD sequences and orthoreovirus S1 sequences constructed using MWHP PCDTW with Euclidean distance and UPGMA clustering. ACE2-binding betacoronavirus sequences are in red boxes and non-ACE2-binding betacoronavirus sequences are in blue boxes. Orthoreovirus sequences are not in boxes.

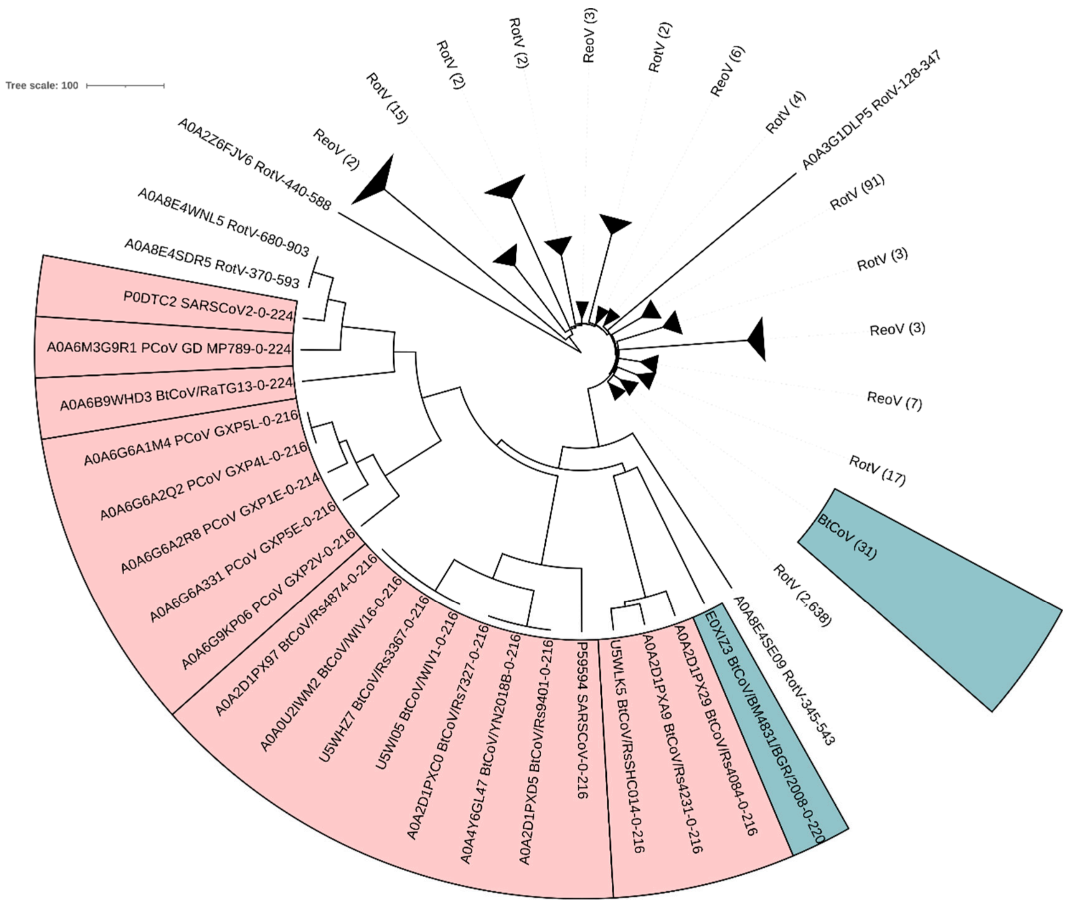

Figure S6: Dendrogram of betacoronavirus spike protein RBD sequences and rotavirus spike protein sequences constructed using MWHP PCDTW with Euclidean distance and UPGMA clustering. ACE2-binding betacoronavirus sequences are in red boxes and non-ACE2-binding betacoronavirus sequences are in blue boxes. Rotavirus sequences are not in boxes..

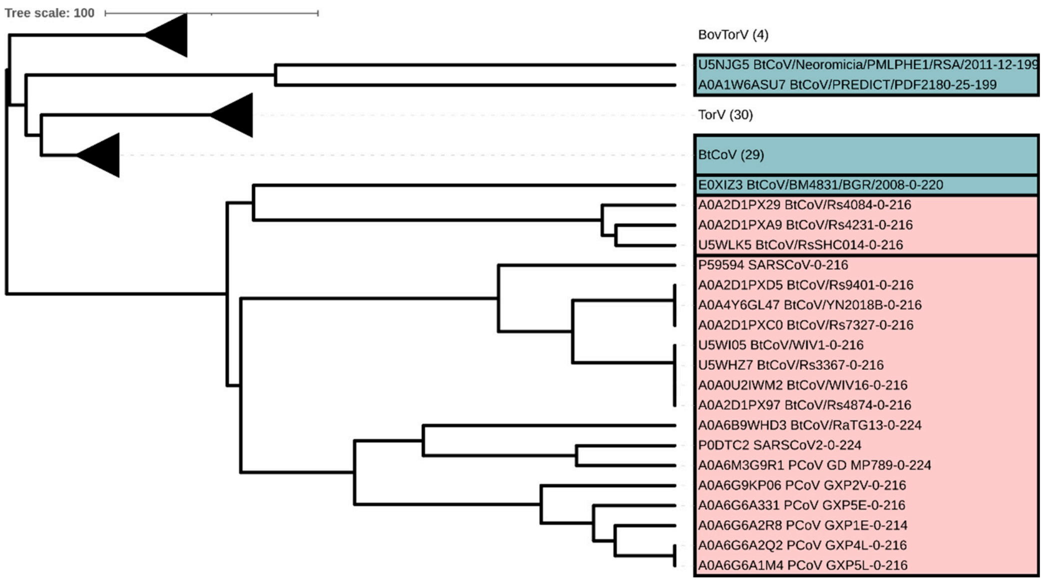

Figure S7: Dendrogram of betacoronavirus spike protein RBD sequences and torovirus spike protein sequences constructed using MWHP PCDTW with Euclidean distance and UPGMA clustering. ACE2-binding betacoronavirus sequences are in red boxes and non-ACE2-binding betacoronavirus sequences are in blue boxes. torovirus sequences are not in boxes.

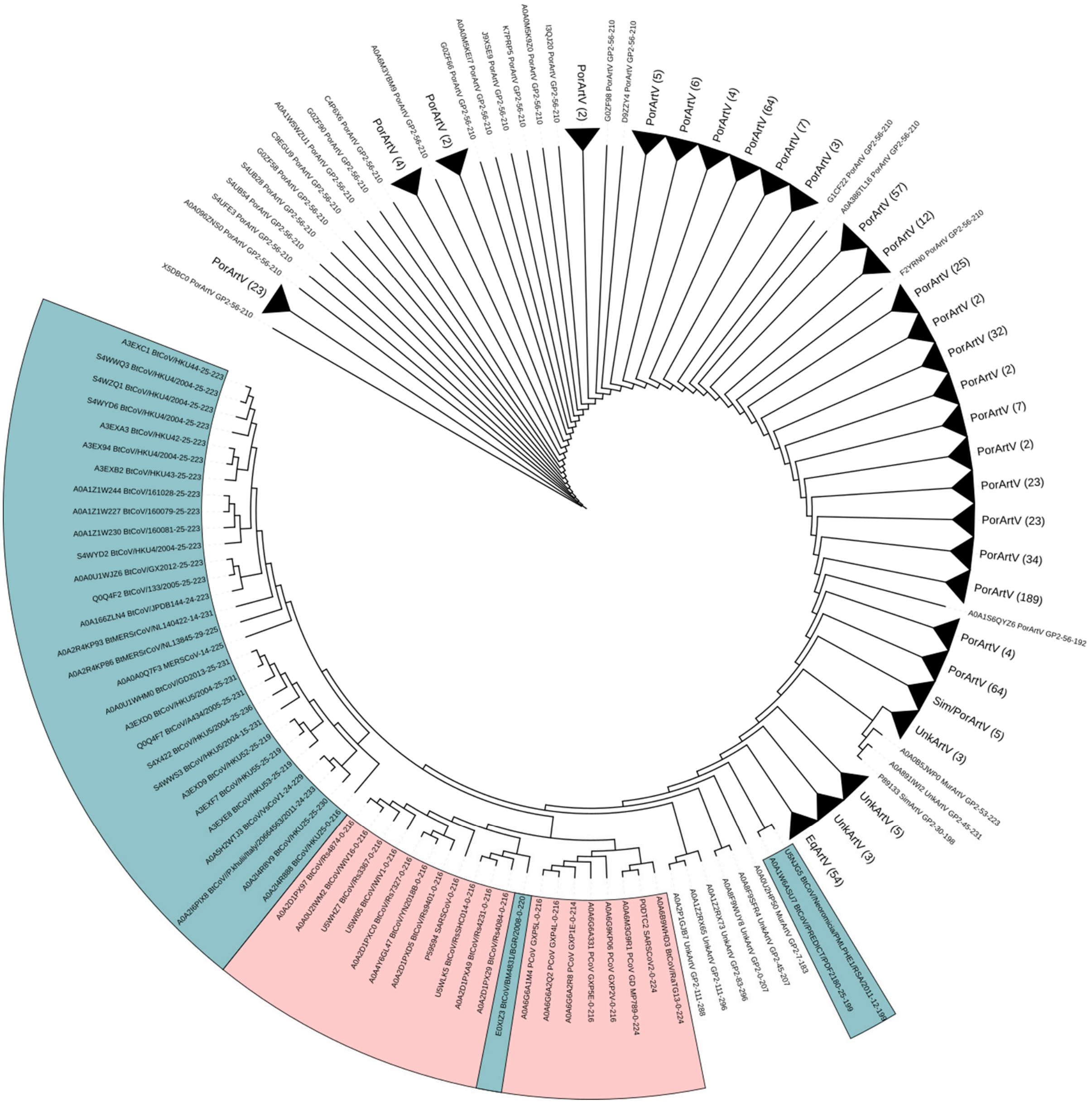

Figure S8: Dendrogram of betacoronavirus spike protein RBD sequences and arterivirus GP2 sequences constructed using MWHP PCDTW with Euclidean distance and neighbor-joining clustering. ACE2-binding betacoronavirus sequences are in red boxes and non-ACE2-binding betacoronavirus sequences are in blue boxes. Arterivirus sequences are not in boxes.

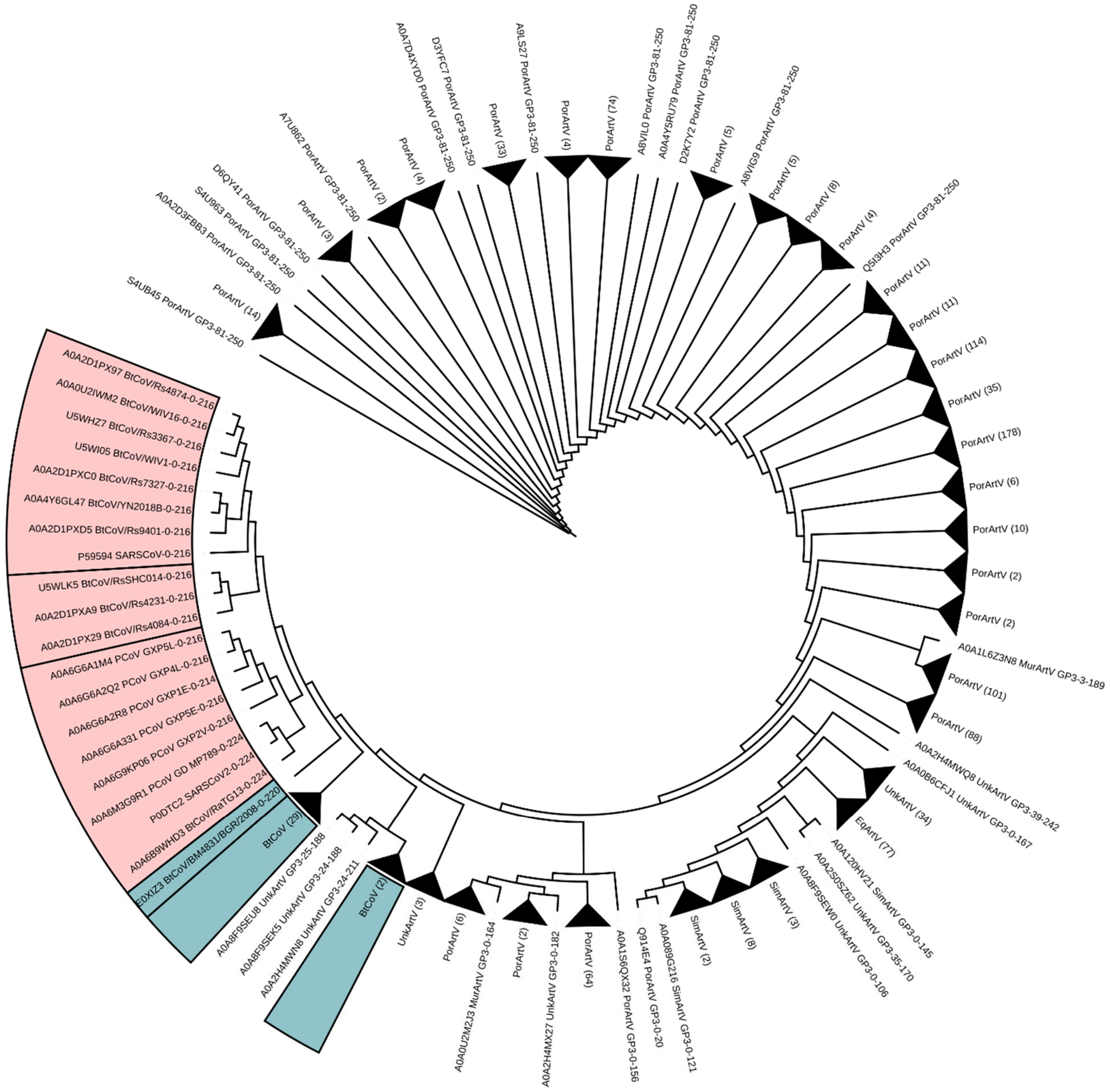

Figure S9: Dendrogram of betacoronavirus spike protein RBD sequences and arterivirus GP3 sequences constructed using MWHP PCDTW with Euclidean distance and neighbor-joining clustering. ACE2-binding betacoronavirus sequences are in red boxes and non-ACE2-binding betacoronavirus sequences are in blue boxes. Arterivirus sequences are not in boxes.

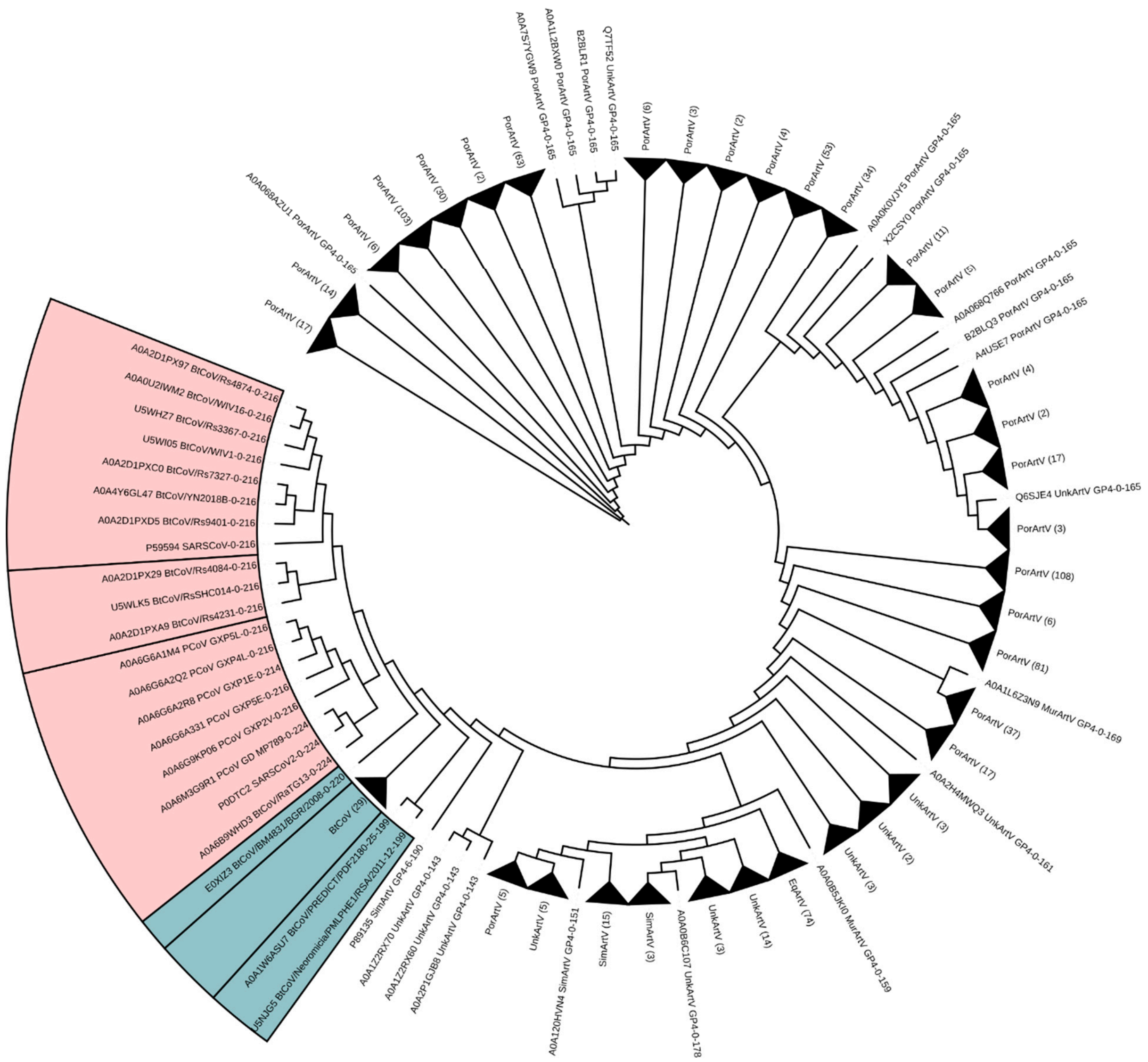

Figure S10: Dendrogram of betacoronavirus spike protein RBD sequences and arterivirus GP4 sequences constructed using MWHP PCDTW with Euclidean distance and neighbor-joining clustering. ACE2-binding betacoronavirus sequences are in red boxes and non-ACE2-binding betacoronavirus sequences are in blue boxes. Arterivirus sequences are not in boxes.

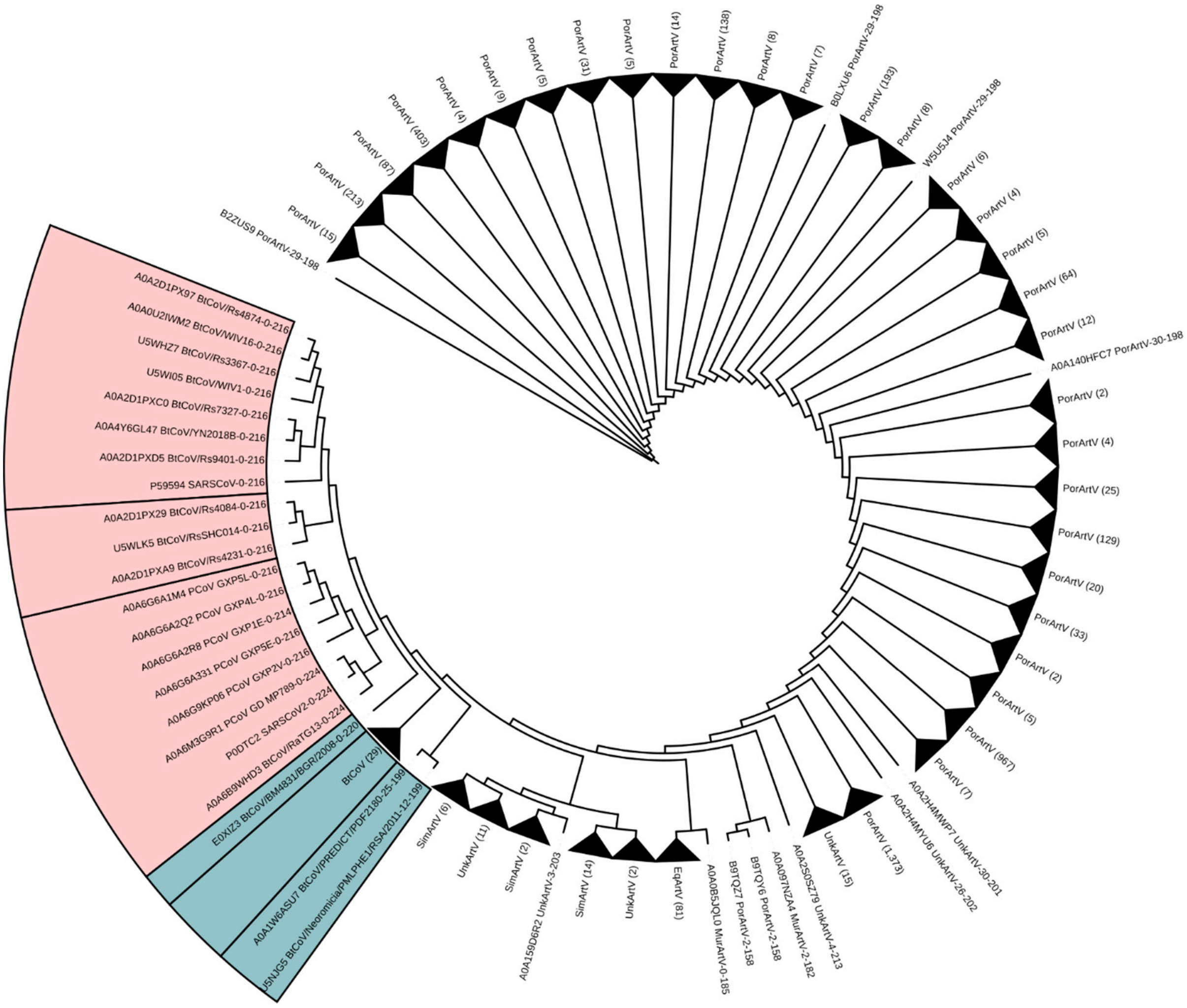

Figure S11: Dendrogram of betacoronavirus spike protein RBD sequences and arterivirus GP5 sequences constructed using MWHP PCDTW with Euclidean distance and neighbor-joining clustering. ACE2-binding betacoronavirus sequences are in red boxes and non-ACE2-binding betacoronavirus sequences are in blue boxes. Arterivirus sequences are not in boxes.

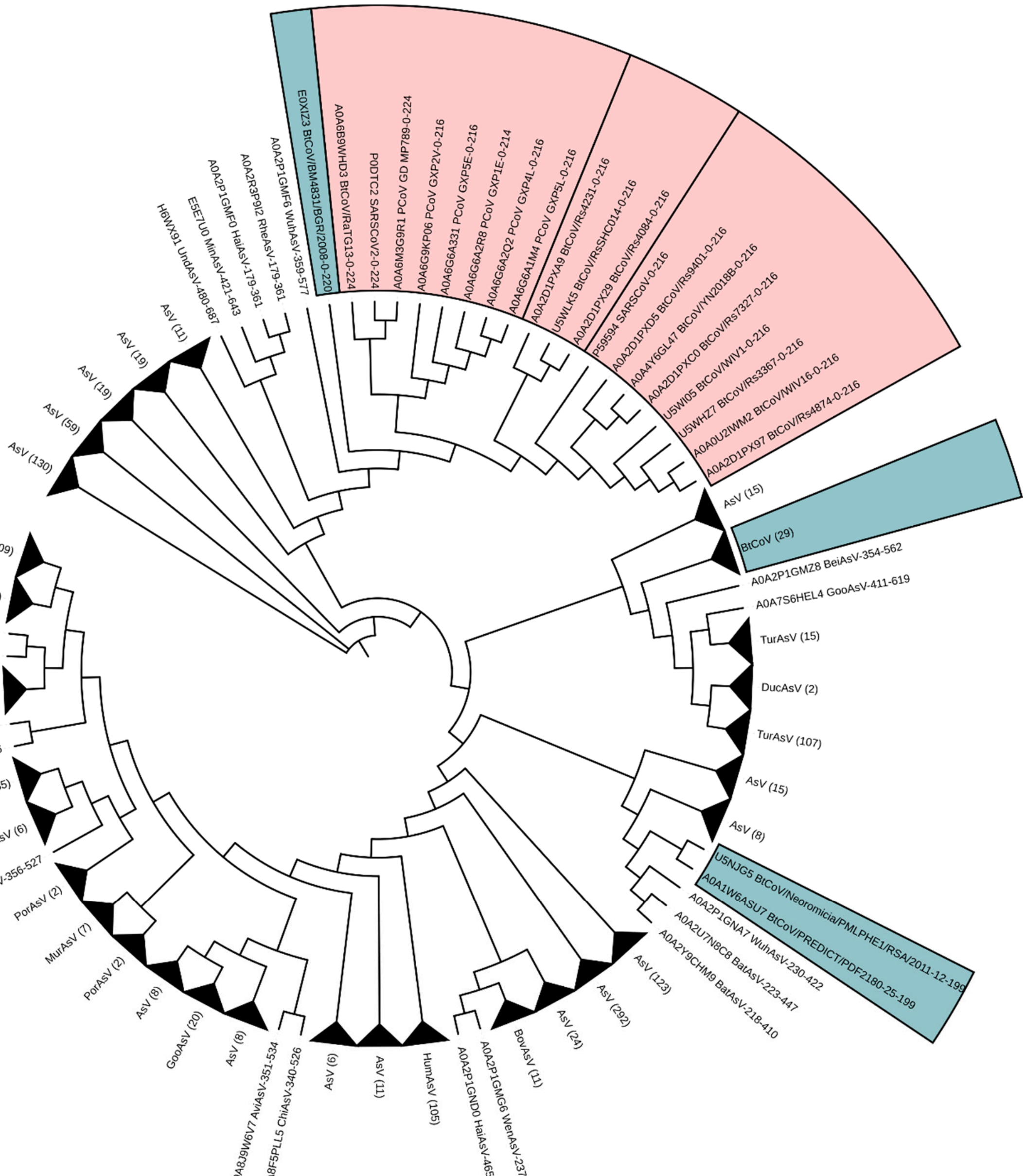

Figure S12: Dendrogram of betacoronavirus spike protein RBD sequences and astrovirus VP90 sequences constructed using MWHP PCDTW with Euclidean distance and neighbor-joining clustering. ACE2-binding betacoronavirus sequences are in red boxes and non-ACE2-binding betacoronavirus sequences are in blue boxes. Astrovirus sequences are not in boxes.

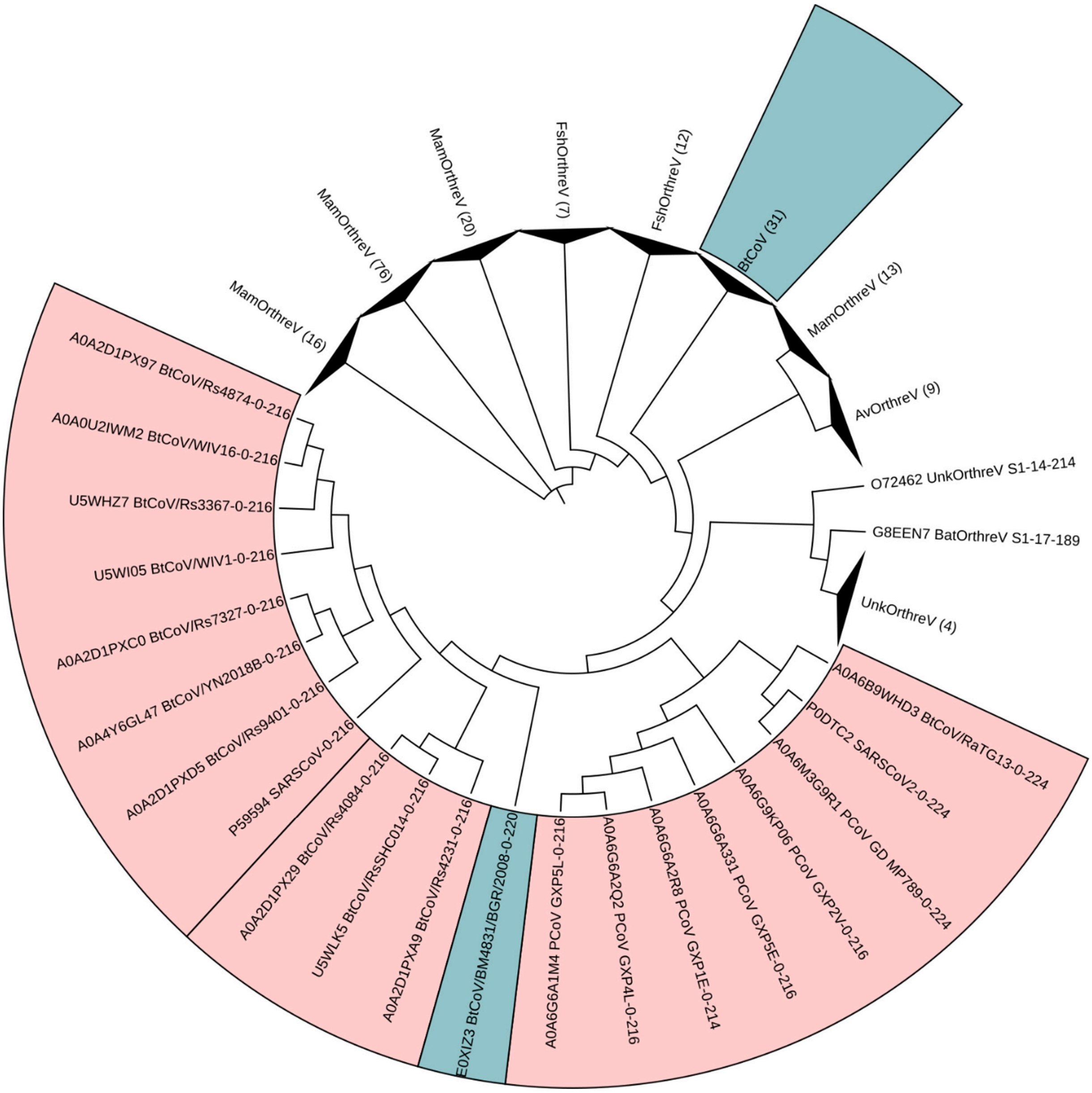

Figure S13: Dendrogram of betacoronavirus spike protein RBD sequences and orthoreovirus S1 sequences constructed using MWHP PCDTW with Euclidean distance and neighbor-joining clustering. ACE2-binding betacoronavirus sequences are in red boxes and non-ACE2-binding betacoronavirus sequences are in blue boxes. Orthoreovirus sequences are not in boxes.

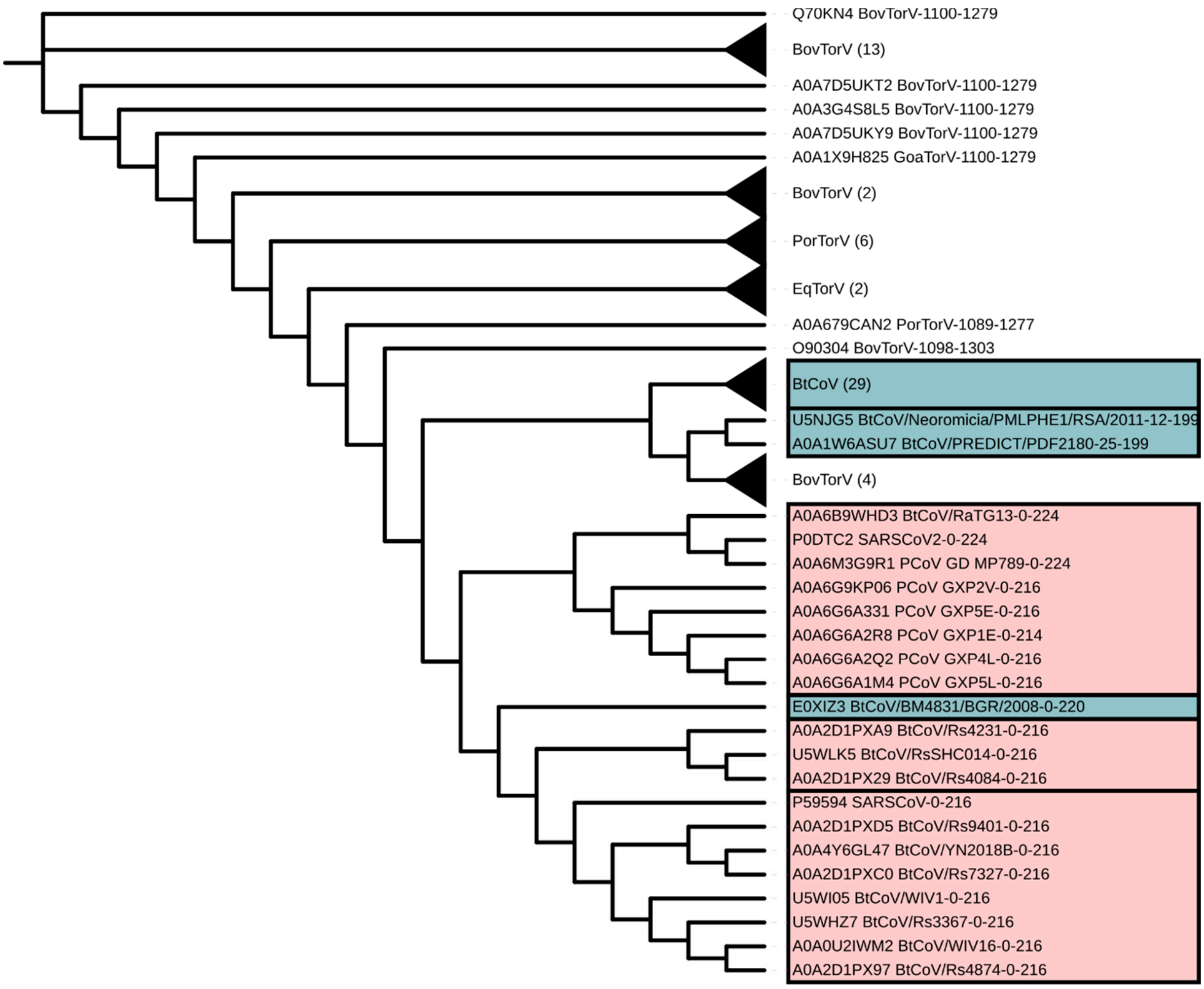

Figure S14: Dendrogram of betacoronavirus spike protein RBD sequences and torovirus spike protein sequences constructed using MWHP PCDTW with Euclidean distance and neighbor-joining clustering. ACE2-binding betacoronavirus sequences are in red boxes and non-ACE2-binding betacoronavirus sequences are in blue boxes. Torovirus sequences are not in boxes.

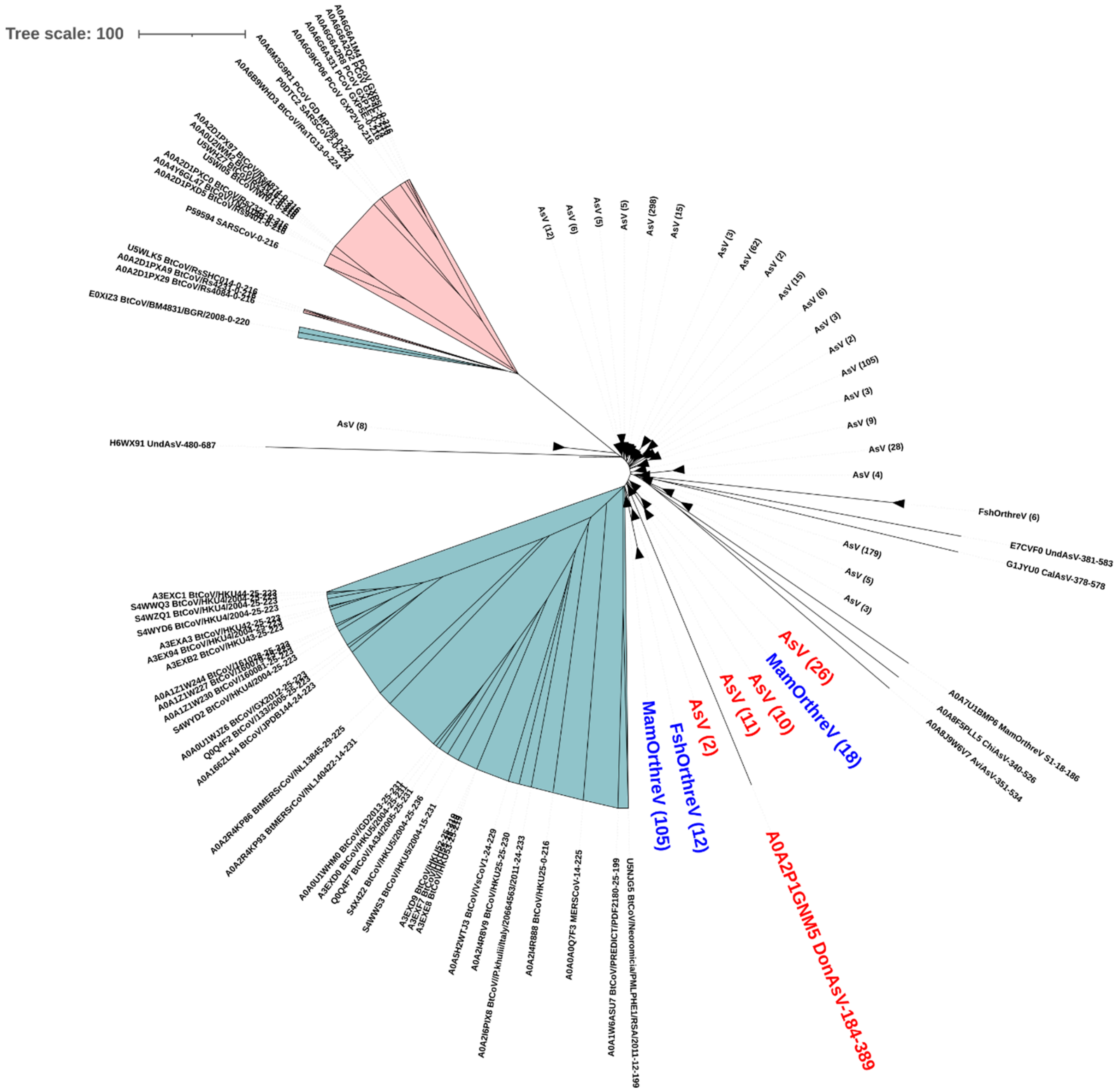

Figure S15: Unrooted UPGMA dendrogram depicting the MWHP PCDTW similarity among betacoronaviruses, astroviruses and orthoreoviruses. Of note is that the structure of the ACE2-binding and non-ACE2-binding betacoronavirus clades is maintained here as in all other analyses herein and that the clades are separated by multiple non-betacoronavirus clades. This along with the star-like nature of the dendrogram suggests a common cenancestor of all of the sequences. Astrovirus and orthoreovirus sequences that cluster more closely with betacoronavirus sequences than with sequences from their own taxa are shown with red and blue labels respectively. ACE2-binding and non-ACE2-binding betacoronavirus clades are highlighted in red and blue respectively.
